# Persistent symptoms and lab abnormalities in patients who recovered from COVID-19

**DOI:** 10.1038/s41598-021-91270-8

**Published:** 2021-06-17

**Authors:** Julian Varghese, Sarah Sandmann, Kevin Ochs, Inga-Marie Schrempf, Christopher Frömmel, Martin Dugas, Hartmut H. Schmidt, Richard Vollenberg, Phil-Robin Tepasse

**Affiliations:** 1 Institute of Medical Informatics, University of Münster, lbert-Schweitzer-Campus 1/Gebäude A11, 48149 Münster, Germany; 2 Department of Medicine B, Gastroenterology and Hepatology, University Hospital Münster, Münster, Germany; 3 Institute of Medical Informatics, University Hospital Heidelberg, Heidelberg, Germany

**Keywords:** Virology, Health care, Medical research

## Abstract

With increasing numbers of patients recovering from COVID-19, there is increasing evidence for persistent symptoms and the need for follow-up studies. This retrospective study included patients without comorbidities, who recovered from COVID-19 and attended an outpatient clinic at a university hospital for follow-up care and potential convalescent plasma donation. Network analysis was applied to visualize symptom combinations and persistent symptoms. Comprehensive lab-testing was ascertained at each follow-up to analyze differences regarding patients with vs without persistent symptoms. 116 patients were included, age range was 18–69 years (median: 41) with follow-ups ranging from 22 to 102 days. The three most frequent persistent symptoms were Fatigue (54%), Dyspnea (29%) and Anosmia (25%). Lymphopenia was present in 13 of 112 (12%) cases. Five of 35 cases (14%) had Lymphopenia in the later follow-up range of 80–102 days. Serum IgA concentration was the only lab parameter with significant difference between patients with vs without persistent symptoms with reduced serum IgA concentrations in the patient cohort of persistent symptoms (*p* = 0.0219). Moreover, subgroup analyses showed that patients with lymphopenia experienced more frequently persistent symptoms. In conclusion, lymphopenia persisted in a noticeable percentage of recovered patients. Patients with persistent symptoms had significantly lower serum IgA levels. Furthermore, our data provides evidence that lymphopenia is associated with persistence of COVID-19 symptoms.

## Introduction

COVID-19 (Coronavirus disease 2019) disease has spread as pandemic, causing more than 1 million global deaths as of November 2020^1^ and shows a multifaceted condition with various symptoms due to various organ manifestations^2–5^. There is also an increasing number of recovered patient cases, which can provide new essential insights on the disease course. While the clinical feature characterization of COVID-19 is well-studied for the disease onset^3,6–8^, follow-up information on persistent symptoms and lab findings is scarce^9^. In addition, most work on symptomatology has not studied clusters of co-occurring symptoms in a single patient. Indeed, a systematic review by Struyf et al.^10^ found that combinations of symptoms were not assessed in a single study. However, as newer publications are quickly emerging, we found one study on frequent symptoms clusters^11^.

This work is a retrospective evaluation of university hospital data of patients, who recovered from COVID-19 and were screened for convalescent plasma donation eligibility. Since patients with comorbidities were excluded, our study provides insights on the healthy population, in which COVID-19 disease course is not well-studied.

Standardized symptom characterization report forms and all free-text elements of discharge letters were added for analyses. This facilitates the extraction of infection-related symptoms, which are not explicitly listed in structured forms. Moreover, details on persistent symptoms and duration to symptom-free phase could be sufficiently extracted in almost all of the cases. As a simple, but highly expressive visualization technique, network analysis illustrates clusters of symptoms and persistent symptoms in one graph-based plot. Blood sampling enabled comprehensive lab-data including whole blood analysis, serum chemistry and immunoglobulins. The combination of detailed symptom duration documentation and raw lab-data enables deep analyses of lab differences—also within the physiological range—in patients with versus without persistent symptoms. By doing this, the study evaluated lab abnormalities including but not limited to general infection parameters, in particular lymphopenia, being discussed as a hot-topic and prognostic factor in disease onset^12–14^**.**

## Methods

### Setting

This is a single center retrospective study in the University Hospital of Münster in Germany. Patients who recovered after confirmed diagnosis of COVID-19 were invited upon an open public call in the Münsterland region of Germany. The primary purpose of this call was to find eligible patients for a plasma donation study^15^. The ethical board of the University of Münster and the physician's chamber of Westphalia-Lippe approved the study protocol (Reference number: AZ 2020–220-f-S)^15^. All included patients provided informed consent for scientific analyses. All methods were performed in accordance with the Declaration of Helsinki and with the relevant guidelines and regulations.

**Figure 1:**
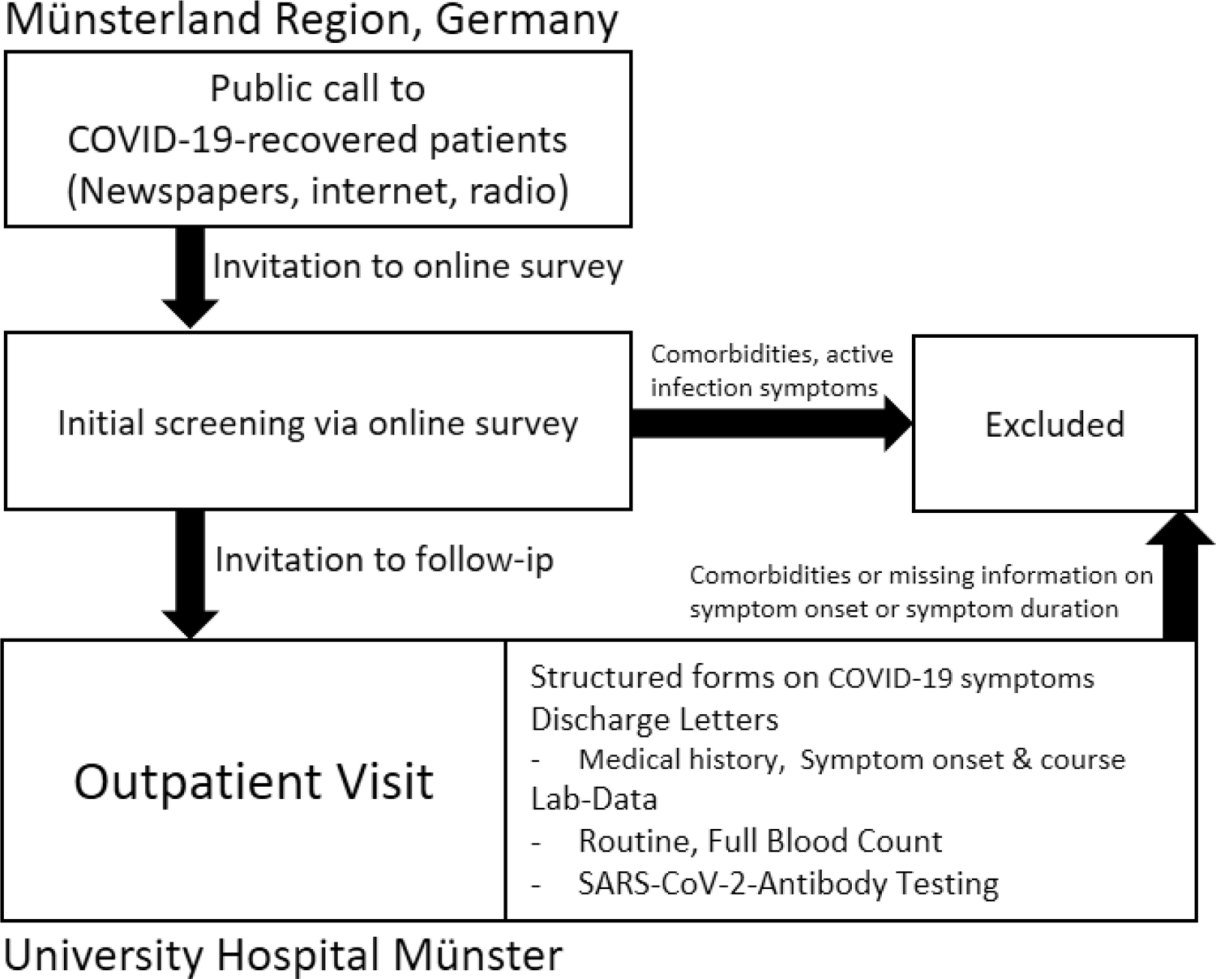
Inclusion of study population.

Figure 1 illustrates the screening process and data collection. Patients with comorbidities or active infection symptoms within the last four weeks were excluded. Final assessment was carried out via outpatient visit at the University Hospital with medical history taking and lab testing. From this stage, further patients were excluded from this study if comorbidities were deemed to be existing or if there was missing information on symptom onset or symptom duration.

### Data extraction

Symptoms were obtained from structured clinical forms and discharge letters. Attending physicians were required to document onset of symptoms, duration and persistence of symptoms. Symptoms were defined to be persistent if they endured for at least 28 days from the onset. Single symptoms and possible combinations were extracted, but not the duration of each single symptom as this was not documented consistently.

Lab values and corresponding reference ranges were queried from the laboratory information system. Discharge letters were examined by a physician with infection-related symptoms being mapped to medical concepts of the standardized forms or added as new symptoms. Symptoms being highly similar were summarized to one medical concept.

### Data analysis

Network analysis of symptoms was conducted using the R-package igraph 1.2^16^ to show frequency and graph-based co-occurrence of onset-symptoms and persistent symptoms. Descriptive statistics were calculated for patient cohort characteristics. Age and gender were compared in the two cohorts with vs without persistent symptoms using 2-tailed t-tests (alpha = 0.05) and Fisher’s exact test, respectively. Lab data was summarized with descriptive statistics and proportions below and above reference ranges. In addition, lab values were compared between the two cohorts using 2-tailed t-tests (alpha = 0.05; adjustment for multiple testing using Bonferroni correction). Calculations were conducted in R, version 4.03.

## Results

### Patient cohort

**Figure 2:**
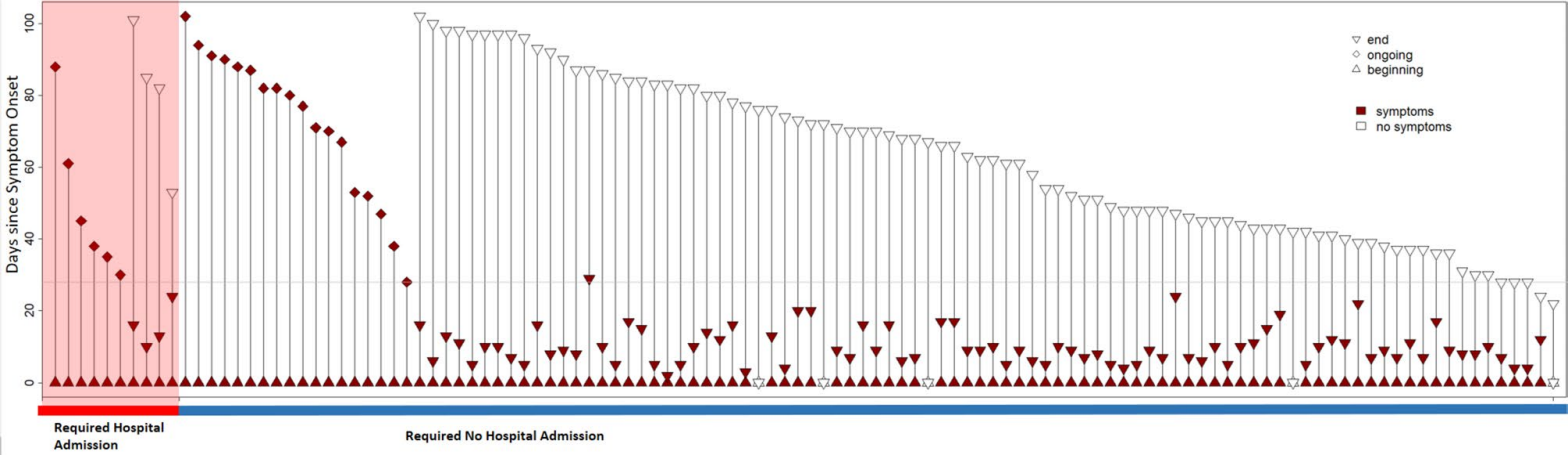
Follow-up day and symptom duration for each patient. Grey horizontal line represents threshold for persistent symptoms (28 days). Five patients were asymptomatic (symptom start and end at day 0).

After participation in the online-survey, 122 patients were screened eligible and attended the outpatient clinic for final assessment during June and September 2020. Six patients were excluded due to insufficient information on symptom duration or comorbidities (n = 116). The age range was 18–69 years, with median of 41 years (IQR 30–54). Table 1 summarizes further cohort details. Comparing the two patient cohorts with (n = 92) vs without persistent symptoms (n = 24), no significant difference can be observed regarding age (t-test, *p* = 0.2497) and gender (Fisher’s exact test, *p* = 0.3415).Follow-up ranged from 22 to 102 days. Figure 2 illustrates follow-up times from symptom onset and symptom durations of all patients.

**Table 1:** Demographic and Clinical Characteristics of patients (n = 116).

Patient Characteristics (n = 116)	Value
Age, median (IQR), y	41 (31–55)
Female sex, no. (%)	17 (15%)
Follow-up time, median (IQR), d	66 (44–82)
Positive SARS-CoV2- antibody testing, no. (%)	70 (80%)
Requiring Hospital admission no. (%)	10 (9%)
With Persistent Symptoms, No. (%)	21%)
- Age, median (IQR), y	34–57)
- Female sex, No. (%)	5 (21%)
Without Persistent Symptoms	79%)
- Age, median (IQR), y	30–54)
- Female sex, No. (%)	12 (13%)

### Symptom analyses

**Figure 3:**
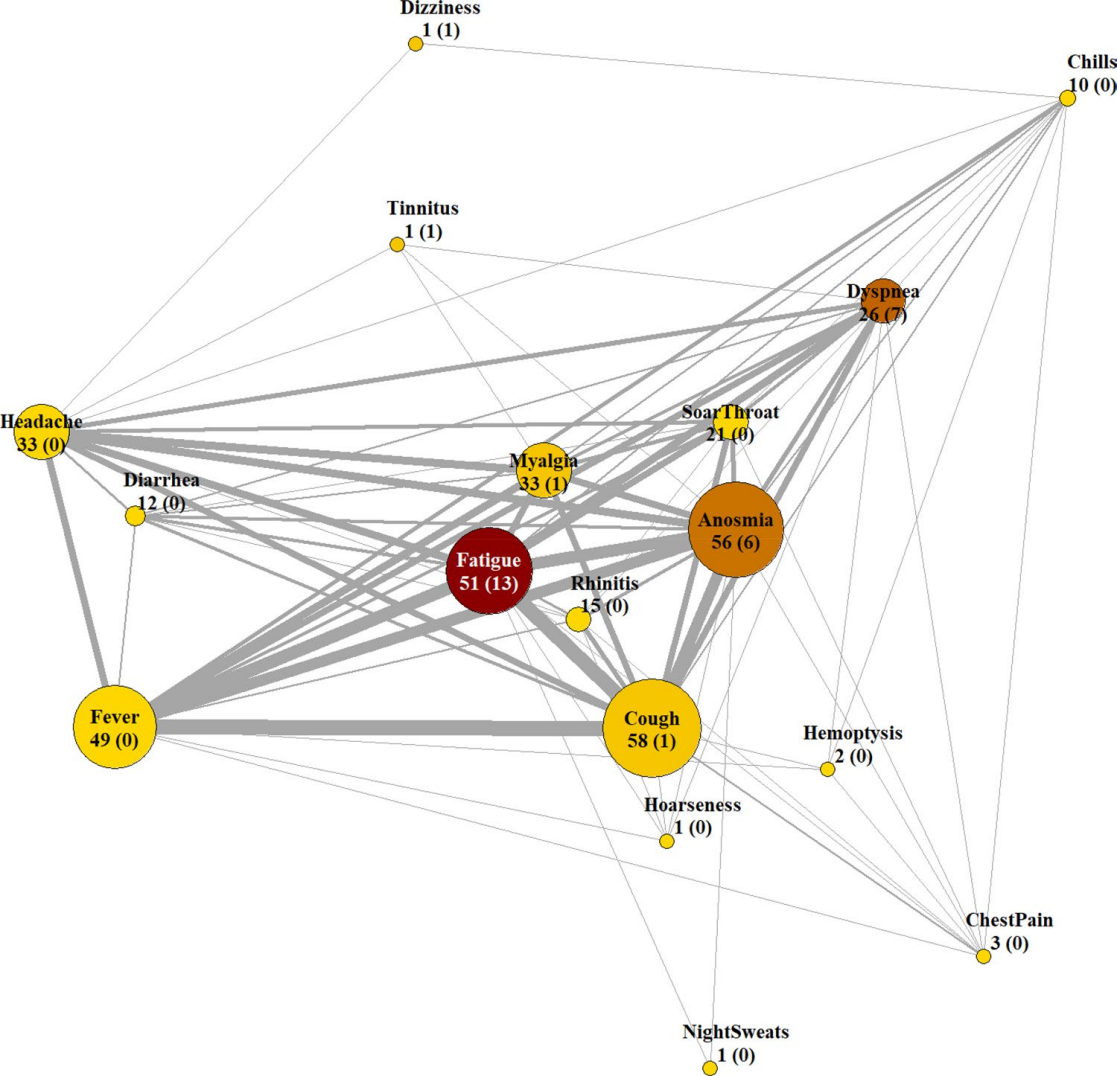
Network analyses of symptom nodes. Node size and node number correspond to the number of patients having that onset symptom. The number in parentheses corresponds to persistent symptoms, which is also indicated by the redness of the symptom node. Thickness of grey edges corresponds co-occurrence of onset symptoms.

After extraction of infection-related concepts in the discharge letters, the concept “Reduced Physical Resilience” was subsumed to “Fatigue”. Myalgia and signs of arthralgia were subsumed to Myalgia. Tinnitus was added as new symptom as it did not occur in the structured standard. Figure 3 shows the network analysis of onset and persistent symptoms. The five most frequent onset symptoms were Cough (50%), Anosmia (47%), Fatigue (45%), Fever (42%), Myalgia (28%) and Headache (28%). Among patients with persistent symptoms (n = 24), the three most frequent persistent symptoms were Fatigue (n = 13, 54%, min = 28d, max ≥ 94d), Dyspnea (29%, min = 25d, max ≥ 102d) and Anosmia (25%, min = 41d, max ≥ 91d). A web-based platform was established to upload anonymous symptoms data to generate and reproduce network analysis based on own or other cohorts^17^.

### Lab abnormalities

**Figure 4:**
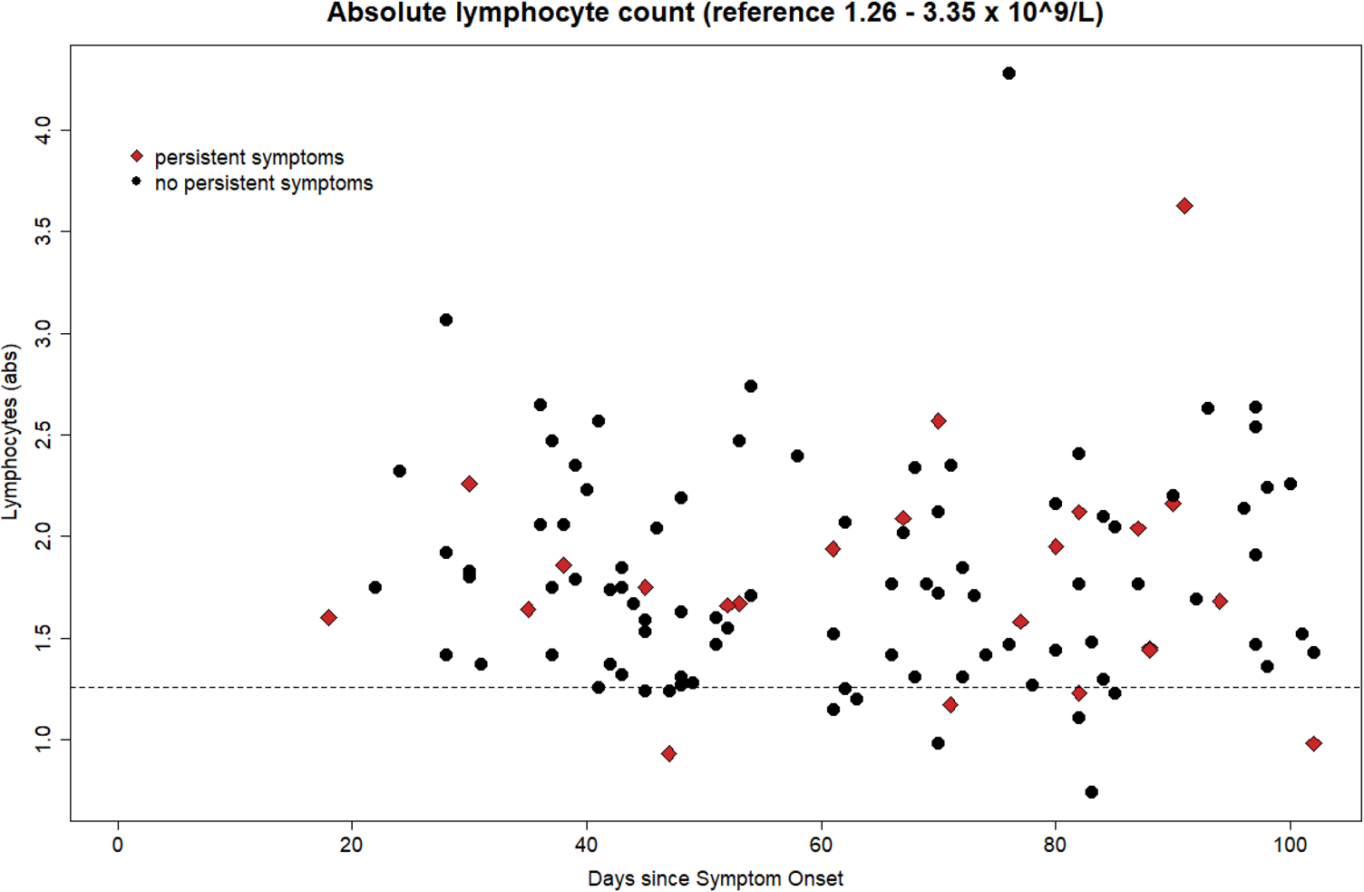
Absolute lymphocyte count over time. Dashed line represents lab-specific lower limit (1.26 × 10^9^/L).

Table 2 summarizes the outcome of all lab measurements, which were available in more than 95% of all patient cases. Increased values above the reference range can be observed in more than 10% of patients for Creatine Kinase, Glucose, GOT, GPT, Potassium, LDH and Platelets count. Decreased values can be observed for eGFR and absolute Lymphocyte count. Lymphopenia was present in 13 of 112 (12%) cases. These cases had results below the lab-specific reference range of 1.26–3.35 × 10^9^/L (follow-up range: 45-102d, median: 70d). Figure 4 details lymphocyte count over all follow-up times for all cases.

**Table 2:** Lab values in alphabetical order for all measures with at least 95% availability among all participants.

Lab-Value (Unit)	All			Comparison PS vs N-PS			
	Normal range %	Below%	Above %	PS, mean (SD)	N-PS, mean (SD)	*p*-value	Adjusted *p*-value
Albumin (g/dL)	98,23	0,00	1,77	4.54 (0.31)	4.59 (0.22)	0,4709	1
Alkal. phosphatase(U/L)	98,23	1,77	0,00	62.87 (15.72)	62.83 (15.91)	0,9922	1
Antithrombin (%)	99,11	0,89	0,00	98.57 (7.55)	97.69 (8.68)	0,6321	1
Basophilic abs. (1000/μL)	93,75	0,89	5,36	0.04 (0.01)	0.04 (0.02)	0,1819	1
Basophilic rel. (%)	94,64	0,89	4,46	0.62 (0.29)	0.74 (0.34)	0,1079	1
Bilirubin total (mg/dL)	95,58	0,00	4,42	0.73 (0.59)	0.56 (0.24)	0,1788	1
CRP (mg/dL)	94,69	0,00	5,31	0.41 (0.04)	0.41 (0.06)	0,7412	1
Creatinine (mg/dL)	100,00	0,00	0,00	0.91 (0.16)	0.92 (0.14)	0,7813	1
Creatine kinase (U/L)	66,37	0,00	33,63	121.78 (64.42)	173.76 (139.93)	0,0110	0,5598
eGFR – CKD EPI (mL/min)	75,22	24,78	0,00	93.57 (4.91)	93.82 (5)	0,8246	1
Total Protein (g/dL)	97,35	1,77	0,88	7.38 (0.34)	7.38 (0.36)	0,9302	1
Eosinoph. abs. (1000/μL)	94,64	2,68	2,68	0.14 (0.08)	0.15 (0.12)	0,5812	1
Eosinoph. relative (%)	95,54	1,79	2,68	2.49 (1.56)	2.68 (2.02)	0,6246	1
Erythrocytes/RBC (Mio./µL)	87,50	2,68	9,82	4.92 (0.45)	5 (0.41)	0,4013	1
Ferritin (µg/L)	83,19	7,08	9,73	191.48 (185.22)	177.03 (140.19)	0,7294	1
Fibrinogen (mg/dL)	95,58	0,88	3,54	311.65 (78.52)	294.34 (48.33)	0,3220	1
Gamma-GT (U/L)	93,81	0,00	6,19	33.04 (25.52)	30.23 (22.04)	0,6319	1
Glucose (mg/dL)	88,60	0,88	10,53	90 (17.68)	90.85 (21.71)	0,8461	1
GOT/AST. (U/L)	81,42	0,00	18,58	28.09 (5.42)	29.1 (8.7)	0,4892	1
GPT/ALT. (U/L)	78,76	0,00	21,24	30.3 (18.04)	31.02 (13.38)	0,8595	1
Hematokrit (%)	93,75	6,25	0,00	42.95 (3.22)	43.1 (2.83)	0,8372	1
Hemoglobin (g/dL)	93,75	5,36	0,89	14.53 (1.23)	14.64 (1.1)	0,6949	1
IgA (mg/dL)	92,98	1,75	5,26	172.17 (57.95)	231.29 (97.25)	0,0004	0,0219
IgG (mg/dL)	98,25	0,00	1,75	1143.2 (185.03)	1113.07 (193.5)	0,4937	1
IgM (mg/dL)	87,72	6,14	6,14	146.26 (192.42)	102.78 (73.43)	0,2979	1
INR (ratio)	100,00	0,00	0,00	0.99 (0.05)	0.98 (0.05)	0,7382	1
Immat. Granulocytes (1000/µL)	99,11	0,00	0,89	0.02 (0.01)	0.02 (0.01)	0,2752	1
Interleukin.6 (pg/mL)	99,12	0,00	0,88	2.09 (0.52)	2.15 (1.1)	0,6824	1
LDH (U/L)	68,14	0,88	30,97	226.74 (46.82)	210.77 (39.87)	0,1431	1
Leukocytes (Tsd./µL)	92,86	7,14	0,00	6.11 (1.36)	5.77 (1.32)	0,2826	1
Lipase (U/L)	93,81	0,00	6,19	40.13 (12.79)	35.32 (14.33)	0,1252	1
Lymphocytes abs. (1000/µL)	86,61	11,61	1,79	1.8 (0.57)	1.81 (0.54)	0,9501	1
Lymphocytes relative (%)	91,07	6,25	2,68	29.62 (7.16)	31.77 (7.34)	0,2084	1
MCH (pg)	91,96	6,25	1,79	29.61 (1.66)	29.31 (1.59)	0,4460	1
MCHC (g/dL)	83,93	3,57	12,50	33.82 (1.01)	33.96 (0.93)	0,5573	1
MCV (fL)	91,07	8,93	0,00	87.58 (3.85)	86.32 (4.07)	0,1771	1
Monocytes abs. (1000/µL)	95,54	2,68	1,79	0.53 (0.18)	0.51 (0.14)	0,5755	1
Monozyten relative (%)	97,32	1,79	0,89	8.89 (2.54)	8.97 (1.91)	0,8864	1
Sodium (mmol/L)	100,00	0,00	0,00	140.74 (1.68)	140.74 (1.68)	0,7060	1
Neutrophilic abs. (1000/µL)	95,54	4,46	0,00	3.6 (1.06)	3.26 (1.05)	0,1686	1
Neutrophilic relative (%)	93,75	3,57	2,68	58.4 (6.65)	55.84 (8.55)	0,1309	1
Part. Thrombopl. Time (s)	91,15	0,88	7,96	34.13 (2.6)	34.19 (3.01)	0,9263	1
Platelets (1000/µL)	83,04	6,25	10,71	265.3 (71.97)	236.55 (48.63)	0,0809	1
Potassium (mmol/L)	74,34	4,42	21,24	4.3 (0.67)	4.35 (0.62)	0,7593	1
Procalcitonin (ng/mL)	100,00	0,00	0,00	0.05 (0.02)	0.05 (0.03)	0,4861	1
Pseudocholinesterase (U/L)	100,00	0,00	0,00	8493.1 (1884.5)	8642.5 (1476.9)	0,7264	1
Sodium (mmol/L)	100,00	0,00	0,00	140.74 (1.68)	140.74 (1.68)	0,7060	1
Thrombin time (s)	100,00	0,00	0,00	17.52 (0.79)	17.19 (0.7)	0,0748	1
TSH (µU/mL)	100,00	0,00	0,00	1.4 (0.69)	1.5 (0.63)	0,5134	1
Urea (mg/dL)	99,12	0,00	0,88	13.43 (3.3)	14.07 (3.45)	0,4223	1

The columns named below and above represent the percentage of patients deviating from the reference range, which encompasses 95% of the healthy population. Comparison PS = Comparison of lab-values in the two cohorts: With vs without persistent symptoms.

The subgroup of 13 lymphopenic cases had 4 patients (31%) with persistent symptoms and 9 patients without persistent symptoms. The remaining 99 cases with normal Lymphocyte count had 19 patients (19%) with persistent symptoms and 80 patients without.

In the late follow-up range of 80–102 days, lymphopenia was still present in 5 of 35 cases (14%, follow-up range: 82-102d, median: 83d). The subgroup of 5 lymphopenic cases had 2 patients with persistent symptoms and 3 patients without persistent symptoms. The remaining 30 cases with normal Lymphocyte count had 8 patients (19%) with persistent symptoms and 22 patients without.

After adjusting for multiple testing for all lab measurements, IgA was the only parameter – also among other Immunoglobulins—that remained significantly different between patients with and without persistent symptoms (see Table 2). It was significantly reduced in patients with persistent symptoms (*p* = 0.0219).

## Discussion

This study found that a noticeable amount of COVID-19-recovered patients still had (n = 24, 21%) persistent symptoms. In 13% of the cases they persisted for 60 days and longer. The three most frequent ones were Fatigue, Anosmia and Dyspnea. These are also listed as long-term symptoms by the National Institute Health and Care Excellence^18^ and CDC as information for health care providers^19^. While Fatigue and Dyspnea are explainable as general infection symptoms and manifestation of the lungs, Anosmia is reported to be a frequent symptom suspecting other pathogenesis than only nasal obstruction^20^. There is growing evidence that the SARS-CoV-2 initiates key cellular processes in the olfactory epithelium^21^.

The results on persistent symptoms are similar to existing follow-up studies: A post-acute- follow-up study in Italy by Carfi et al.^9^ evaluated Fatigue and Dyspnea but not Anosmia as persistent symptoms (mean follow-up 60.3 days). A follow-up study by Carvalho-Schneider et al.^22^ reported the identical set of our most common persistent symptoms (follow-up at day 30 and 60, non-critical COVID-19 patients). Comparing to a study by Garrigues et al.^23^ with a mean of 110.9 days, Fatigue and Dyspnea were the most common, followed by Loss of Memory. These findings support a common symptom set regardless of patient case severity. However, as with many symptom-related infection studies, signs of neuro-degenerative or mental health disorders could be under-reported. They were not documented in our study as they were not reported by the patients and not part of the structured forms.

Regarding duration of persistent symptoms, our results are significantly different: The post-acute setting in Carfi et al. observed 87.2% of patients having persistent symptom with mean follow-up of around 60 days. Calvao-Schneider et al. 2020^22^ reported that 66% of patients experienced symptoms at day 60^22^. The difference should be explained by our comorbidity-free and relatively young population (median age: 41 ys). Moreover, the majority of the population in Calvao-Schneider et al. had an initial hospitalization rate of 35% vs. 9% in our study.

The main strength of our study is the integration of detailed symptoms duration and raw lab-data, which enabled not only assessment of out of range values but also differences within the physiological range. The study results show that some of the infection-related lab values remained out of range during follow-up. In particular, lymphopenia—being frequently discussed as a result of direct or indirect viral interactions with lymphocytes^12–14^—remained in a considerable part of our study population, even beyond 90 days of follow-up (see Fig. 4). Our subgroup analyses showed that patients with lymphopenia experienced more frequently persistent symptoms. To the best of our knowledge, this is the first COVID-19-related study on the duration of lymphopenia along with other routine lab values going beyond 30 days. Further follow-up studies should assess lymphopenia with additional lab analysis to understand the pathogenetic details of lymphocyte-virus interaction.

There are many drivers being discussed to elucidate on the COVID-19-specific pathogenesis related to lymphopenia. SARS-CoV-2 induced apoptosis in ACE2-receptor expressing lymphocytes was found as one possible mechanism that could lead to lymphopenia in COVID-19^24^. The COVID-19 associated “cytokine storm” syndrome is considered another possible driver of lymphocyte apoptosis^25,26^. Both mechanisms can possibly lead to rapid reduction of lymphocyte counts during acute infection. In our study we found evidence that lymphopenia persists for weeks after recovery from acute disease. There is rising evidence of bone marrow damage due to SARS-CoV-2 infection of hematopoietic stem cells^27^. For SARS-CoV lymphopenia due to thymus suppression has been proposed^28^. Both bone marrow damage and thymus suppression seem possible mechanisms that may lead to persisting lymphopenia after recovery from disease. A recently published study provided evidence of increased mortality in COVID-19 patients during 6 month after recovery from disease. Besides increased risk of cardiovascular, gastrointestinal and thrombotic complications, rates of infections were found to be significantly increased^29^. An association of persistent lymphopenia with increased rates of infections due to a relevant immunosuppressive status is hypothetic and needs further research.

The comparison of patients with vs without persistent symptoms did not show any differences of lab values except for one: serum concentration of total IgA antibodies. In comparison, there were no significant changes regarding total IgG, IgM and all other lab-tests in Table 2. Secretory IgA is an important factor of mucosal immunity for neutralization of toxins and pathogenic microbes^30,31^. While a high level of serum IgA and SARS-CoV-2-specific IgA on diagnosis can initially correlate with disease severity^32,33^, our findings suggest that high levels during or after recovery is associated with a decrease in persistent symptoms. In this regard, IgA may play a more important role than other immunoglobulin types. As this study only reports on lab-value correlations, further research can complement our findings regarding causality.

The principal limitation of this study is the retrospective single-center design with symptom assessment not being objective and being prone to recall bias. Moreover, the different follow-up times also lead to different time distances between last day of symptoms and the actual lab-assessment. As some lab-values return to normal levels over a certain time, some lab tests and association with symptom persistence could have faded out and thus failed to show significance in our analysis.

## Conclusion

Our study results could confirm the main persistent symptoms of COVID-19. Lymphopenia persisted in a noticeable percentage of recovered patients beyond 30 days after disease diagnosis. Patients with persistent symptoms had significantly lower serum IgA concentration, which requires further studies to understand detailed pathogenesis.
